# Developmental Landscape of Potential Vaccine Candidates Based on Viral Vector for Prophylaxis of COVID-19

**DOI:** 10.3389/fmolb.2021.635337

**Published:** 2021-04-15

**Authors:** Rajashri Bezbaruah, Pobitra Borah, Bibhuti Bhushan Kakoti, Nizar A. Al-Shar’I, Balakumar Chandrasekaran, Da’san M. M. Jaradat, Munir A. Al-Zeer, Saeid Abu-Romman

**Affiliations:** ^1^ Department of Pharmaceutical Sciences, Faculty of Science and Engineering, Dibrugarh University, Dibrugarh, India; ^2^ School of Pharmacy, Graphic Era Hill University, Dehradun, India; ^3^ Department of Medicinal Chemistry and Pharmacognosy, Faculty of Pharmacy, Jordan University of Science and Technology, Irbid, Jordan; ^4^ Faculty of Pharmacy, Philadelphia University, Amman, Jordan; ^5^ Department of Chemistry, Faculty of Science, Al-Balqa Applied University, Al-Salt, Jordan; ^6^ Department of Applied Biochemistry, Institute of Biotechnology, Technical University of Berlin, Berlin, Germany; ^7^ Department of Biotechnology, Faculty of Agricultural Technology, Al-Balqa Applied University, Al-Salt, Jordan

**Keywords:** COVID-19, vaccines, viral vector, ChAdOx1-S, Ad5-nCoV, MERS-CoV

## Abstract

Severe acute respiratory syndrome coronavirus 2, SARS-CoV-2, arose at the end of 2019 as a zoonotic virus, which is the causative agent of the novel coronavirus outbreak COVID-19. Without any clear indications of abatement, the disease has become a major healthcare threat across the globe, owing to prolonged incubation period, high prevalence, and absence of existing drugs or vaccines. Development of COVID-19 vaccine is being considered as the most efficient strategy to curtail the ongoing pandemic. Following publication of genetic sequence of SARS-CoV-2, globally extensive research and development work has been in progress to develop a vaccine against the disease. The use of genetic engineering, recombinant technologies, and other computational tools has led to the expansion of several promising vaccine candidates. The range of technology platforms being evaluated, including virus-like particles, peptides, nucleic acid (DNA and RNA), recombinant proteins, inactivated virus, live attenuated viruses, and viral vectors (replicating and non-replicating) approaches, are striking features of the vaccine development strategies. Viral vectors, the next-generation vaccine platforms, provide a convenient method for delivering vaccine antigens into the host cell to induce antigenic proteins which can be tailored to arouse an assortment of immune responses, as evident from the success of smallpox vaccine and Ervebo vaccine against Ebola virus. As per the World Health Organization, till January 22, 2021, 14 viral vector vaccine candidates are under clinical development including 10 nonreplicating and four replicating types. Moreover, another 39 candidates based on viral vector platform are under preclinical evaluation. This review will outline the current developmental landscape and discuss issues that remain critical to the success or failure of viral vector vaccine candidates against COVID-19.

## Introduction

Novel corona virus disease 2020 or COVID-19, caused by the virus severe acute respiratory syndrome coronavirus 2 (abbreviated as SARS-CoV-2), has become an universal outbreak and primary health concern, since its emergence by the end of 2019 in Wuhan, China (Carlos et al., 2020; Guo et al., 2020). The natural host of the virus origin was suspected to be the bats due to higher similarity in genomic sequences, possibly transmitted to humans via an unknown intermediate, further leading to human-to-human transmission through droplets or direct communication (Carlos et al., 2020; Guo et al., 2020). Following swine flu (2009), Ebola in West Africa (2014), polio (2014), Zika (2016), and Ebola in Democratic Republic of Congo (2019) outbreaks, the World Health Organization (WHO) has acknowledged COVID-19 as the sixth public health emergency of global concern (Yoo, 2019). At the time of writing this review, 54, 771, 888 laboratory-confirmed cases and 1,324,249 deaths, owing to this pandemic, had been reported (WHO, 2020f).

Coronavirus is a positive-sense, single-stranded, RNA viruses of the family *Coronaviridae*; which may affect a broad host range exhibiting symptoms ranging from very mild rhinorrhea to severe fatal illness (Dhama et al., 2020; Kotta et al., 2020). Polygenetic sequencing and evolutionary investigations demonstrated that SARS-CoV-2 is a beta-coronavirus, which displayed 96.2%, 79.5%, and 50% sequence identity with previously identified bat CoV RaTG13, severe acute respiratory syndrome coronavirus (SARS-CoV), and Middle East respiratory syndrome coronavirus (MERS-CoV), respectively (Jin et al., 2020). Like SARS-CoV, SARS-CoV-2 uses angiotensin-converting enzyme receptor 2 (ACE2) as the entry receptor and manifests similar acute respiratory syndromes (Lee et al., 2006; Iwasaki and Yang, 2020). Despite higher resemblance with the SARS-CoV genome sequence, it shows different transmissibility and diagnosis procedures because of the mutational changes, i.e., existence of a peculiar furin-like cleavage site in the receptor-binding domain of the spike (S) proteins (Coutard et al., 2020). Of importance, continuous mutations in the S protein–encoding genes have allegedly enhanced the virulence capacity of the virus (Q. Li et al., 2020). The replication cycle of SARS-CoV-2 has a close resemblance with SARS-CoV. After transmission of the virus into the human body, it interacts with the host cells *via* the envelope S proteins. The primary host target receptor for SARS-CoV-2 is angiotensin-converting enzyme 2 (ACE2). Binding of the virus to the ACE2 receptor is mediated by RBD, and fusion of the virus with the host plasmalemma is mediated by the S2 domain (Yu et al., 2020). Acid-dependent proteolysis by serine 2, cathepsin, and other proteases initiate the trimer cleavage of S protein, which exposes the fusion peptide. The fusion peptide inserts into the host cell membrane and produces the antiparallel 6-helix bundle that results in membrane fusion and releases the viral genome into the cytoplasm of the host cell (Ashour et al., 2020; Badgujar et al., 2020). The uncoated RNA of the virus with ORF1a and 1b scrambles viral proteases–processed polyproteins to produce some nonstructural proteins, which produces replication–transcription complex (RTC) inside a double-layered vesicle. RTC undergoes continuous replication and produces about six–nine subgenomic RNAs (Borah et al., 2021a). These RNAs act as the mRNA template for the translation of structural and accessory proteins. Then S, E, and M proteins undergo translation and insert themselves within the endoplasmic reticulum. Moreover, these proteins produce the mature virus particles by assembling with N protein–encapsidated viral genome within the endoplasmic reticulum–golgi intermediate compartment. Following these, the virion is transported to the plasmalemma and released by exocytosis (Borah et al., 2021b).

The WHO treatment guidelines recommended isolation of the COVID-19-suspected patients to provide supportive care including immunomodulatory therapy, oxygen therapy, and antibiotics as per requirement (WHO, 2020a). At present, no Food and Drug Administration (FDA)–approved antiviral or immunomodulatory agents are available for the management of the SARS-CoV-2 infection. However, some promising antiviral agents (viz., remdesivir, ritonavir, and lopinavir alone or in conjunction with interferon-β, favipiravir, etc.), natural products, and some repurposed drugs are under investigation and will be tested through clinical trials (Borah et al., 2020; Dhama et al., 2020; Coronavirus COVID-19, 2020). Without any clear indications of abatement, the disease has become a major healthcare threat across the globe, owing to high prevalence, prolonged incubation period, and absence of existing drugs or vaccines. In order to safeguard the whole global population from continuing danger of morbidity and mortality from SARS-CoV-2, it is crucial to develop and administer an adequate safe and effective vaccine (Awadasseid et al., 2021). In the past decades, many attempts have been undertaken to produce vaccines for human coronaviruses (CoVs) like SARS and MERS, but no approved antiviral therapy or vaccines exists to date. The majority of clinical options available for COVID-19 management are based on prior expertize with the treatment of MERS and SARS-CoV (Cyranoski, 2020). Intensive global R&D efforts have been carried out following the identification of the genetic sequence of SARS-CoV-2 aiming to develop an effective vaccine against the disease (Yadav et al., 2020). However, this can be a long-term practical approach (Zhang et al., 2020b). The use of genetic engineering, recombinant technology and other computational techniques has resulted in several potential candidates for COVID-19 vaccines being produced (Chen et al., 2020; Lundstrom, 2020). As per the WHO, till January 22, 2021, 64 vaccine candidates are under clinical development and 173 candidates are under preclinical evaluation (WHO, 2021). The range of vaccine technology platforms being evaluated, including nucleic acid (DNA and RNA), peptides, virus-like particles, recombinant proteins, live-attenuated viruses, viral vectors (replicating and non-replicating), and inactivated virus approaches, are striking features of the vaccine development landscape for COVID-19 (WHO, 2020b). The majority of vaccines undergoing clinical and preclinical trials involve next-generation vaccine platforms, such as vaccine based on nucleic acid, antigen-presenting cells, or viral vectors (Rauch et al., 2018; van Riel and de Wit, 2020). Succeeding the triumph of Ebola vaccine (Commissioner, 2020), viral vectors can get the appraisal as a valid tool for delivering vaccine into a host cell for generation of antigens that can be tailored to arouse a robust immune response (Bouard et al., 2009; The Scientist, 2020). Furthermore, the promising results of the viral vector–based vaccine candidates against MERS/SARS infections had added a value to this development. This article encompasses brief summary of viral vectors as a potential vaccine development platform, highlights ongoing advances in designing (SelectScience COVID-19 Vaccine, 2021) vaccine candidates based on viral vectors, and also sheds light on the issues that remain critical to the success or failure of viral vector vaccine candidates against COVID-19.

## Viral Vector–Based Vaccine

**FIGURE 1:**
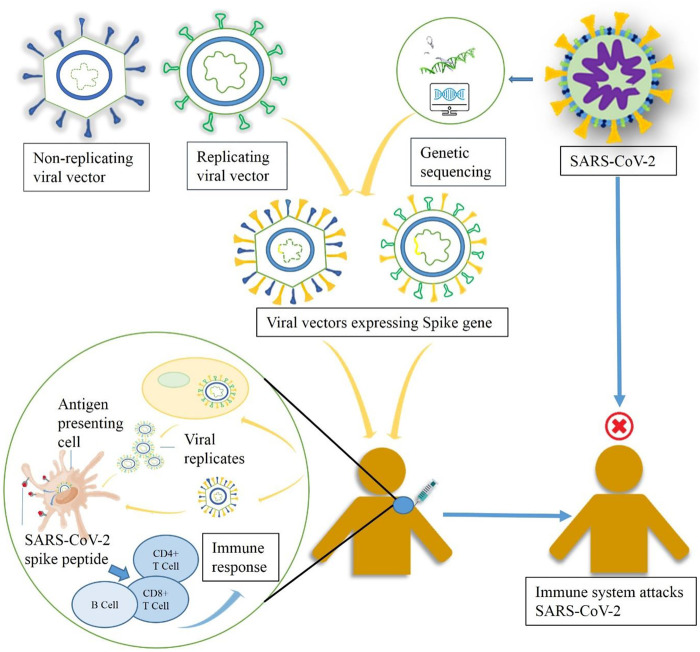
A schematic diagram representing the working principle of replicating and nonreplicating viral vector–based vaccine candidates expressing the spike protein of SARS-CoV-2.

The development of viral vector–based vaccine is a specialized area of *in vivo* gene therapy. Gene therapy aims to rectify genetic diseases by permanent replacement of a missing or damaged gene with transgene product, introduced *via* an immune-tolerated carrier vehicle (Ura et al., 2014). Vaccines, on the other hand, aim to provoke a strong immune response against pathogens via introduction of the same pathogenic antigen, along with the supportive inflammatory responses shown by the delivery vehicle. Despite their different aims, gene therapy and vaccines use recombinant viral vectors as a common platform to express therapeutic transgene product and immunogenic antigen, respectively (Ertl, 2016). Followed by extensive research work, the concept of viral vectors has been modified from gene therapy in 1980. Viral vectors are produced by replacing the viral gene with pathogenic transgene or antigen; following administration, the antigen is shuttled into host cells leading to expression of immune responses against that particular pathogen (Rogers et al., 1973; Bouard et al., 2009). In the majority of viral vector–based vaccines, a single dose is adequate for producing a prophylactic action owing to expression of endogenous antigens that stimulate both humoral and cellular immunity (van Riel and de Wit, 2020). Another advantage of viral vector–based vaccines are highly specific targeted gene delivery, improved gene transduction efficiency, enhanced safety and efficacy, and easy large-scale manufacturing (Cai et al., 2020; Creative Biolabs, 2021). Since the explosion of viral vectors as vaccines’ development platform, a large number of viral vector–based vaccines have been permitted for veterinary medicine. In 2011, Imojev (vaccine against Japanese encephalitis) was the first approved viral vector–based vaccine for clinical use in humans (Rollier et al., 2011). As a basis for establishing vaccines based on viral vectors, a wide variety of viruses have been used; for example, some commonly used viruses are adenoviruses (Ad), poxviruses, adeno-associated viruses (AAV), parvoviruses, lentivirus, togaviruses, measles viruses, etc. (Ramezanpour et al., 2016). This platform involves viral vectors that can either be replicating (replication-competent), often attenuated, or nonreplicating (replication-defective) (Robert-Guroff, 2007). The replicating vector vaccines infect the host cells, which thereafter give rise to vaccine antigens as well as new viruses that may infect more cells and express immunogenicity. However, nonreplicating vector vaccines are capable of infecting the host cells and produce vaccine antigens but fails to produce new virus particles (van Riel and de Wit, 2020). The storage temperature for viral vector–based vaccines is in-between +2 and +8°C (SelectScience). Figure 1 provides a schematic representation of the development of immune responses against SARS-CoV-2 by replicating and nonreplicating viral vector–based vaccines. A brief description of the commonly used viral vectors is provided below.

### Adenovirus (Ad)

Adenovirus, which is known to cause respiratory tract infections, is a nonenveloped DNA virus having a double-stranded genome of about 30–40 kb that is enclosed by an icosahedral capsid. It was the first DNA virus to go in diligent therapeutic development with a tremendous interest, mainly because of its high transduction efficiency, genetic stability, wide range of viral tropism, and high expression level of transgenes. In human, 57 serotypes of adenovirus were identified with different tropism mechanism, that are grouped into seven species (A–G) (Seymour and Fisher, 2011; Crystal, 2014; Ura et al., 2014; Lee et al., 2017; Rauch et al., 2018). Ad vaccines are developed by replacement of the genomic regions-early transcript 1 A and early transcript 1 B (E1A and E1B) by transgenes. These modifications eliminate the replicating ability of the virus; thereby, they are considered as replication-defective vectors. Additionally, E3 and E4 genes are often deleted to avoid the abolition of Ad-infected cells by the immune system and to prevent the leaky expression of the inserted transgene, respectively (Wold and Toth, 2013; Rauch et al., 2018). A common method of production of Ad vector involves transfection of plasmid of Ad vector into E1–complementing cell lines (HEK 293 cells), where they infect the cells and undergo replication; newly replicated vectors are collected and subjected to purification using ultracentrifugation (Ura et al., 2014; Ramezanpour et al., 2016). Depending on the employed serotype, Ad vectors can induce both cell-mediated and antibody-mediated immunity with a variation in immune response (Humphreys and Sebastian, 2018). Replication-deficient human Ad serotype (Ad5) can be easily produced in high titers, so they possess a great attraction as a gene delivery vector (Tan et al., 2013; Ura et al., 2014). Nevertheless, preexisting immunity of the immunized person may obstruct the clinical use of this virus. In order to overcome this limitation, adenoviral vectors had been developed from nonhuman origin, for example, the chimpanzee virus–derived vector ChAd63. Moreover, selection of rare serotypes with reduced risk in human (viz. Ad26 or Ad 35) is an alternative way to overcome the resistance (Rauch et al., 2018; Buchbinder et al., 2020).

### Poxviruses

Poxviruses are the most extensively studied viral vectors. In 1978, vaccinia virus (VACV, a *Poxvirus* family member) was found to be successful for eradicating small pox virus (Jenner, 1988). It is a huge, complex, and enveloped double-stranded DNA virus. The size of the DNA genome is approximately 190 kb in length, and it accepts about 25 kb of antigen (Jacobs et al., 2009). Numerous highly attenuated VACV strains are available for use in humans and animals, which includes both replication-competent and replication-deficient strains (Ura et al., 2014). One of the most commonly used, well-characterized VACV strains is a replication deficient-attenuated VACV called modified vaccinia virus Ankara (MVA), which is constructed by the removal of 15% vaccinia genome by sequential passaging through chicken embryo fibroblasts (Sutter and Staib, 2003). Some other examples of replication deficient strains are New York–attenuated vaccinia virus or NYVAC (derived from Copenhagen strain of vaccinia), ALVAC (avipox vectors: canarypox), and FPV (fowlpox) (Franchini et al., 2004; Parrino and Graham, 2006). Vaccines based on vaccinia virus shows high-transgene expression and thus can produce a robust immunity against antigens. Moreover, they induced innate immunity facilitated by the inflammasome and Toll-like receptors (TLRs). One limitation of vaccinia vaccine is that the efficacy may be affected by preexisting immunity (Cooney et al., 1991; Ura et al., 2014).

### Measles Virus (MV)

Measles virus belonging to *Paramyxoviridae* family is an enveloped RNA virus, with a single-stranded, nonsegmented, negative-sense genome of approximately 16 kb. MV vaccine is produced by repetitive serial passage of infectious virus *via* various cell lines that results in a live-attenuated and replication deficient virus. The process undergoes numerous mutations that makes MV vaccine genetically stable; moreover, retrogression to pathogenicity has never been detected (Zuniga et al., 2007). Additionally, the virus is unable to merge into the host genome, and thermostability of the virus has been established by lyophilization. With all these advantages, MV vaccine shows extremely durable immunization induced by both humoral- and cell-mediated immune response (Ovsyannikova et al., 2003; Tangy and Naim, 2005). Unlike adenoviral vector (where T-cell–mediated response is dominated by CD8^+^ phenotype), MV shows CD4^+^ dominated T-cell–mediated response, and that may be a consideration for vaccine generation (Rauch et al., 2018). MV is a valuable promising vaccine delivery system because of efficient transgene expression aptitude and low production cost, and most significantly, MV genome has the capability of stable insertion of more than 5,000 nucleotides (Zuniga et al., 2007; Loessner et al., 2012). Owing to its ability to withstand relatively large transgenes, multipathogen or multivalent MV–based vaccines can be produced (Lauer et al., 2017). Furthermore, antivaccine efficacy of MV vaccine is not compromised by the vector immunity (Ramsauer et al., 2015).

### Sendai Virus (SeV)

Sendai virus is a single-stranded, negative-stranded, nonsegmented, enveloped RNA virus, which is a member of *Paramyxoviridae* family (Nakanishi and Otsu, 2012). SeV is found to be nonpathogenic in humans, but it causes bronchopneumonia in mice (Ura et al., 2014). SeV is found to have high resemblances with the human parainfluenza type-1 virus (hPIV-1), and thus, activity of the SeV vector is affected by preexisting host immunity against hPIV-1. Cell entry and tropism of the SeV genome are mediated by two enveloped glycoproteins, namely, hemagglutinin–neuraminidase (HN) and fusion glycoprotein FO (F). Deficiency of these proteins generates replication-defective virus and advances vector’s safety (Ura et al., 2014). In the first generation of SeV vectors, replication-competent vectors were produced by installing exogenous cDNA in the full-length SeV genome. However, for practical applications, replication-defective SeV vectors were produced by transfecting the packaging cell with a genome in which F gene has been replaced with transgene. The SeV vector can transduce both dividing as well as nondividing cells. It contains viral genome and RNA–dependent RNA polymerase in their cytoplasm, which ensures genotoxic advantages of the virus. It also confirms fast gene expression following an infection. Its transgene capacity (i.e., 3.4 kb) is low compared to the other viral vectors (Nakanishi and Otsu, 2012; Ura et al., 2014).

### Adeno-Associated Virus (AAV)

Adeno-associated virus is a member of *Parvoviridae* family, which is a small, nonpathogenic, nonenveloped, single-stranded DNA virus (Romano, 2005). The virus shows low immunogenicity as it contains only two genes that can be replaced with transgene, and for replication, it is dependent on helper virus functions. The genome size of AAV is 4.7 kb, and once infected a human cell, it integrates with human genome at a specific site on 19q chromosome. The integration includes the inverted terminal repeats (ITR) and *Rep* region at both terminals of the viral genome, providing a high level of expression. Furthermore, the virus shows wide tropism and can infect both dividing and nondividing cells (Johnson et al., 2005; Liniger et al., 2007; Ura et al., 2014). There are 12 AAV serotypes available to be used in humans, of which AAV2 is mostly used in clinical and preclinical practices. More than 100 serotypes of AAV are found in various animal species. Each serotype has own receptor and tissue specificity (Xiao et al., 1999). Recombinant AAV vectors are produced by replacing *Cap* and *Rep* regions between the ITRs with transgenes. Following these modifications, AAV vectors cannot integrate into the host genome (Ura et al., 2014). AAV vector has low–titer production efficiency in comparison to other viral vectors. To compensate for this limitation; large-scale, highly efficient production has been developed (Urabe et al., 2002; Ura et al., 2014). After transducing the host cell, AAV provoke innate immune response and produce interferon (IFN) α/β. In Kupffer cells, induction of TLR9– and TLR2–dependent cytokine expression was also observed. AAV produces mild humoral- and cell-mediated immune response. Besides, immunogenicity is affected by preexisting immunity and neutralizing antibodies, thus AAV vector–based vaccines are rarely used in clinical trials. Several recombinant, randomly mutant or hybrid recombinant AAV are produced to improve the efficacy of AAV for vaccine development (Cai et al., 2020).

## Viral Vectors as a Promising Platform for Some of the Deadliest Diseases

### Smallpox

Smallpox disease caused by variola virus was a contagious disease that claimed millions of lives till the time of its eradication (Fenner et al., 1988; Parrino and Graham, 2006). The worldwide eradication of variola virus was a tremendous success that was achieved by the introduction of VACV vaccine. In 1796, Edward Jenner, an English doctor, has experimented the use of cowpox virus against small pox; then, it was followed by a good number of experiments in 1801, where Jenner published his discoveries. Soon after that, vaccination became widely accepted, and at some point in the 1800s, the cowpox virus has been replaced with vaccinia virus (VACV) (CDC, 2019). In order to have cross-protection against *Variola* virus, VACV was used for nearly 2 decades until the obliteration of smallpox in the late 1970s. Though the origin of VACV remains unidentified, it is mostly related to horsepox virus (Tulman et al., 2006). Most of the vaccines were developed on living animal’s skin like calves, sheep, rabbits, and buffalos. During the eradication program, various vaccinia virus strains, such as Lister (Elstree), the New York City Board of Health (NYCBH), Copenhagan, and Ankara MVA strain had been used for the development of vaccines (Parrino and Graham, 2006; Jacobs et al., 2009). As vector, Jennerian vaccine viruses (viz., Dryvax, Lister, and Copenhagan) signify the first generation of VACV vaccines. Second generation of VACV includes tissue culture–adapted Jennerian. Since the time of eradication, continued research on several strategies such as genetic engineering of immunomodulatory proteins–encoded viral genes, and serial passage in an alternative host have developed several modified VACV vaccines with enhanced safety profile. At present time, third and fourth generation of VACV attenuated by passage in an alternate host and genetic engineering, respectively, are considered for stockpiling in opposition to a potential revive of smallpox by bioterrorism. The propensity of the VACV genome to accumulate new genetic material allows researchers to develop new vaccines against a broad variety of contagious diseases (Jacobs et al., 2009).

### Influenza

Influenza (the flu) caused by the virus influenza is a contagious disease. Moreover, influenza is a source of several respiratory tract infections, and it causes annual epidemics. Though the infection is self-limiting, it may develop severe complications in certain patient groups that may be lethal (Medina and García-Sastre, 2011). Several vaccine formulations are available against circulating influenza strains. Viral vectors are also used for influenza vaccine production. Soon after the success of vaccinia virus for eradicating small pox disease, recombinant vaccinia viruses were designed as a viral vector vaccine to express antigens of influenza virus. Smith et al. generated the first viral vector expressing the influenza hemagglutinin (HA) gene, and that vaccine was able to produce protective immune response in animal models (Smith et al., 1983). After this, various recombinant viral vector vaccines expressing different influenza virus proteins like NP, M1, PA, NA, PB1, and PB2 were designed (Smith et al., 1987; de Vries and Rimmelzwaan, 2016). Modified vaccinia virus Ankara (MVA) vector is produced by sequential passing of chorioallantois vaccinia virus Ankara through fibroblast of chicken embryo (Mayr and Munz, 1964). Other attenuated poxviruses such as NYVAC (Kyriakis et al., 2009), raccoonpox (Kingstad-Bakke et al., 2012), canarypox (Minke et al., 2007), and fowl-pox (Taylor et al., 1988) have been used as viral vectors for the development of influenza vaccine candidates. Furthermore, several other viruses like recombinant herpes virus, alpha virus, vesicular stomatitis virus, baculovirus, Newcastle disease virus, and adenovirus vectors were also used to generate vaccine against influenza virus (de Vries and Rimmelzwaan, 2016).

### Malaria

Malaria, affecting billions of people globally, is a significant factor of mortality and morbidity. Viral vectors are outstanding vector vehicle for malarial antigens. For the transmission of malaria antigens classic viral vectors like adenovirus, alphavirus, and poxvirus vectors have been successfully used (Li et al., 2007). In a phase I/II clinical trial, a multi-stage, multi-antigen, poxvirus–vectored vaccine candidate NYVAC-Pf7 had showed low-titer immune response against malaria infection (Ockenhouse et al., 1998). Other attenuated poxviruses like MVA tend to be less efficient in priming immune response, but is best at immune response boosting. However, in conjunction with other vectors or protein/DNA–based vaccines, a VACV vector is likely to be helpful as heterologous prime-boosting regimens (Schneider et al., 1998). For example, heterologous prime boost immunization with Chimpanzee adenovirus 63 and modified vaccinia Ankara encoding thrombospondin–related anonymous protein (ChAd63 MV A ME-TRAP) found to be safe and immunogenic vaccine regimen against malaria, though protection efficacy is not significant (Ogwang et al., 2015; Bliss et al., 2018; Tiono et al., 2018). New emerging vectors like measles virus, yellow fever virus, and vesicular stomatitis virus (VSV) offer supplementary opportunities for designing malaria vaccine candidates (Li et al., 2007). A preclinical investigation of a malaria vaccine candidate based on recombinant measles viruses (rMV) expressing PbCS (circumsporozoite antigen of *Plasmodium berghei*) and PfCS (circumsporozoite antigen of *Plasmodium falciparum*) demonstrated induction of high-antibody response in mice that remain for at least 22 weeks post-prime. The vaccine candidate also confirmed rapid development of cellular immunity (Mura et al., 2019).

### Cancer

In the field of cancer research, the aim of cancer vaccine is to induce strong and durable effective immune response against self-antigens which are tumor-associated antigens (TAAs) and tumor-specific antigens (TSAs) (Guo et al., 2013). Various strategies have been proposed to develop therapeutic cancer vaccines, among which viral vector platform is showing promising results. IMLYGIC, the first oncolytic viral therapy approved in the US, is based on a genetically modified herpes simplex type-1 virus (FDA, 2019). Poxviral vectors or its prototype VACV are among the mostly used viruses in the production of cancer vaccines (DeMaria and Bilusic, 2001). A randomized phase II trial of PROSTVAC (a poxviral prostate-specific Ag targeting vaccine) in men suffering from metastatic castration-resistant prostate cancer demonstrated an increase in overall patients survival. However, a recent phase III study concluded that PROSTVAC induce T-cells that have the capability of tumor infiltration but the T-cell–mediated immune response does not translate into therapeutic benefit. The study results suggested that poxvirus can be a promising platform when used with different antigen targets, in combination with checkpoint inhibitor, or in other disease settings (Gulley et al., 2019). Adenoviruses, when used in animal models (Lundstrom, 2017), have shown potential therapeutic effects for gastric cancer, hepatic carcinoma, prostate (Ekblad and Halldén, 2010), ovarian (Matthews et al., 2009), and brain cancer (Fu et al., 2010). Shapira et al. demonstrated that adenoviral vectors encoding a pro-apoptotic PUMA gene regulated by RAS-responsive elements (Ets/AP1) can suppress the growth of cancer cell with KRAS mutation (Shapira et al., 2017). In addition to the above-mentioned vectors, several other viral vectors including AAV, lentivirus, Newcastle disease virus, measles virus, rhabdo viruses, and baculo viruses are being engineered for cancer vaccine development (Lundstrom, 2017).

### Ebola

Ebola hemorrhagic fever caused by Ebola virus is one of the deadliest viral disease affecting humans and nonhuman primates worldwide. In response to 2014 outbreak, Ebola has been acknowledged as a public health emergency of international concern by WHO (2020c). Responding to this emergency, vaccine development against Ebola was accelerated. Several replicating and nonreplicating viral vectors such as alphavirus, flavivirus, adenovirus, vaccinia virus, and paramyxovirus have been used for vaccine development. Of these, two prime candidates have emerged, namely, the chimpanzee adenovirus–based vaccine (ChAd3-EBO-Z) (Tapia et al., 2015) and the recombinant vesicular stomatitis virus–based vaccine (rVSVΔG-ZEBOV-GP) (Piszczatoski and Gums, 2020). Another candidate consisting of an adenovirus type 26 vector vaccine encoded with glycoprotein of Ebola (Ad26.ZEBOV), and a modified vector vaccine of vaccinia Ankara (MVA-BN-Filo) have shown promising results (WHO, 2018) and is in phase III clinical trial (ClinicaltrialsNCT04556526, 2020L). Recently, the European Medicines Agency has proposed the marketing authorization of the vaccine Ad26.ZEBOV/MVA-BN-Filo to be given in the European Union (EMA, 2020). rVSVΔG-ZEBOV-GP or Ervebo is a recombinant, live, replication-competent, attenuated vaccine comprising a backbone of the vesicular stomatitis virus (VSV), which is modified to express a Zaire Ebolavirus glycoprotein to produce the neutralizing immune response of a host to the Ebola virus. Ervebo is given as a single dose. After tremendous research work, Ervebo was approved by the FDA as the first vaccine against Ebola. The approval was published on December 19, 2019, and the medication is under postmarketing surveillance by the European Medical Agency (EMA) (Piszczatoski and Gums, 2020).

### AIDS

The human immunodeficiency virus (HIV), the causative organism of acquired immunodeficiency syndrome (AIDS), weakens the immune system against several infectious conditions, including some types of cancer (WHO, 2020d). Over 30 HIV/AIDS vaccine candidates with positive response in nonhuman primate models have progressed to clinical trial either alone or in combination (Ross et al., 2010). Among which viral vectors is the most promising way to deliver HIV immunogens for induction of cellular immunity to HIV. Additionally, prime-boost strategies of viral vectors had shown promising results. Many viral vector–based HIV vaccine candidates are under clinical and preclinical investigation, including adenovirus, poxvirus, alpha virus, and adeno-associated virus, in addition to combination of viral vectors encoding HIV gene (Sauter et al., 2005). For the first time, RV-144 in a phase III efficacy clinical trial in Thailand had shown that HIV infection can be prevented by vaccination. This trial assessed the effectiveness of four priming immunizations of ALVAC-HIV (vCP1521) in combination with two booster injections of a recombinant gp120 subunit vaccine (AIDSVAX B/E) (Rerks-Ngarm et al., 2009; Pantaleo et al., 2010). Another phase I clinical trial had demonstrated that upon single administration, MVA-B, a poxvirus-based HIV/AIDS vaccine candidate triggers a robust, polyfunctional, long lasting T-cell response against HIV-1infection in human (Gómez et al., 2011). A preclinical study had confirmed that dendritic cell vaccine based on lentiviral vector can supress the replication of HIV in improved mice model (Norton et al., 2019).

## History of Viral Vector–Based Vaccines Development Against SARS and MERS-CoV

Around the year 1960, the first endemic coronavirus infection was recognized. Till date, total seven coronavirus infections including SARS-CoV-2 has been identified, among which four (viz. HCoV-229 E, HCoV-NL63, HCoV-0C43, and HCoV-HKU1) were known to cause endemic by triggering minor diseases like common cold or flu in immune-compromised populations (Corman et al., 2018). Another two epidemic coronavirus infections emerged around 2002 and 2012, known as SARS-CoV and MERS-CoV, respectively, also exhibited flu-like symptoms and fatal acute respiratory infections (Badgujar et al., 2020). Isolation and phylogenetic examination of the newly emerged coronavirus (SARS-CoV-2), responsible for causing COVID-19, showed similarity with SARS-CoV virus; thus, the new virus is referred to as SARS-CoV-2 (Lu et al., 2020). So far, no vaccines are available against human coronavirus infections, though dozens of coronavirus vaccine candidates are being evaluated; subsequently, the epidemic of SARS-CoV and MERS-CoV is in preclinical and early clinical studies. Spike (S) glycoprotein was used as a target antigen in most of the cases. However, one SARS-CoV and four MERS-CoV vaccine candidates were advanced to early clinical trial, and those are based on S protein. It has been assumed that, in case of SARS-CoV, owing to fast disappearance of the virus, only one vaccine has completed phase I trial and other two trials were withdrawn. Notably, three of these potential MERS/SARS vaccine candidates are from viral vector platform, while the other two are DNA-based vaccine (Zhang et al., 2020a). Human Ad-vector, chimpanzee Ad-vector, and MVA-vector have been employed for designing BVRS-GamVac (ClinicaltrialsNCT04130594, 2019), ChAdOx1MERS (MERS001) (ClinicaltrialsNCT03399578, 2018a), and MVA-MERS-S (ClinicaltrialsNCT03615911, 2018b) vaccines, respectively. Guo et al. reported that single intramuscular immunization of mice with recombinant human adenoviral (type 5 or 41) vector vaccine encoded with full-length S protein of MERS-CoV can produce mucosal T-cell–mediated immune response and systemic neutralizing antibodies. However, T-cell–mediated immunity is not observed in case of intragastric route administration (Guo et al., 2015). Intriguingly, the vector rAd5 encoded with shorter S1 extracellular domain of S protein had manifest slightly stronger neutralizing antibody responses than full-length S protein. This signifies the effect of immunofocusing (Kim et al., 2014). Hashem et al. (2019) had established that rAd5 expressing CD40–targeted S1 fusion protein (rAd5-S1/F/CD40 L) provide a total protection against MERS-CoV in the hDPP4 transgenic mice model and also prevent pulmonary perivascular hemorrhage (Hashem et al., 2019). Currently, phase I and phase II clinical trials are in progress for BVRS-GamVac (a human Ad-vector–based vaccine candidate against MERS-CoV) with the aim to assess safety and immunogenicity (NCT04130594).

Chimpanzee adenovirus due to deficiency of preexisting immunity and attractive safety profile represents a good alternative to human adenoviral vector (Dicks et al., 2012). METRS001, a ChAdOx1 vaccine candidate encoding MERS-CoV S protein, has recently completed phase I, non-randomized, dose-escalation, uncontrolled, open-label trial, and at all tested doses, was found to be safe and well-tolerated. Furthermore, a single dose was capable of eliciting both cellular and humoral immunity against MERS-CoV (Folegatti et al., 2020).

The full-length S protein–encoded recombinant MVA also represents a promising vaccine candidate for MERS-CoV, owing to its better immunogenicity, high safety, and protective profile for MERS-CoV (Song et al., 2013; Volz et al., 2015). A MVA-based vaccine candidate, MVA-MERS-S, is currently under phase-I human trial, where the safety and immunogenicity of the vaccine will be investigated in healthy adults (NCT03615911). Recently, another report of recombinant MVA vaccine expressing the S protein of MERS-CoV confirmed safety and immunogenicity against MERS-CoV upon intramuscular administration in a phase I clinical trial (Koch et al., 2020). Nevertheless, MVA encoding highly conserved N protein along with S protein of MERS-CoV found to provoke CD8^+^ T-cell response, but the protecting efficacy is not yet investigated (Veit et al., 2018). Apart from these, MERS-CoV’s vaccine based on Newcastle disease virus (NDV) can induce neutralizing antibodies in Bactrian camels and BALB/c mice (LIU et al., 2017). Another live-attenuated measles virus–based vaccine candidate, namely, MV_vac2_-MERS-S(H) encoded with MERS-CoV spike glycoprotein, had shown multifunctional cellular immunity in preclinical study (Bodmer et al., 2018). Currently, a list prepared by WHO showed eight viral vector–based SARS-CoV vaccine candidates under preclinical investigation (WHO, 2020e).

In the current set-up, the preceding understandings of coronavirus vaccine development such as immunogenic response, antigen, challenges while using animal models, adjuvants, and route of administration may add some supplementary role in the rapid development of a vaccine against COVID-19. Viral vector–based vaccine candidates are second-generation vaccines in antiviral vaccine development strategies and more beneficial compared to first generation vaccine because they vaccinate the live virus into a nonvirulent vector by recombining the antigenic protein component of pathogenic virus. Thus, it imitates the possible natural pathogenic contamination, followed by humoral and cellular immunity (Yong et al., 2019; Badgujar et al., 2020; Zhang et al., 2020a; Zhang et al., 2020b).

Regalado et al. have mentioned some explanations for the shortage of reliable and commercial vaccines against SARS and MERS-CoV. With MERS-CoV, vaccine development, it is likely to be delayed due to the shortage of appropriate and productive small animal model during preclinical study. Another cause may be the lack of interest to invest in a vaccine for a comparatively low and geographically centralized disease as compared to other more chronic and global infectious diseases (HIV, measles, and tuberculosis). On the other hand, SARS-CoV cases ceased to be reported in 2004, and thus, further investigation in SARS-CoV’s vaccine was assumed to be futile (Padron-Regalado, 2020).

## COVID-19 and Viral Vector–Based Vaccine Platform

In current scenario, the ongoing COVID-19 pandemic has greatly accelerated the requirement of a massively producible, safe, and efficient vaccine for SARS-CoV-2. In such instance, vector-based vaccines have come as the front-runner. As illustrated by successful eradication of smallpox (Ura et al., 2014), FDA approval of Ervebo (CDC, 2020), and other promising vaccine candidates against various infectious diseases, viral vectors offer themselves as an attractive platform for the development of vaccines against COVID-19. Additionally, the lesson acquired from the viral vector–based vaccine development strategies for MERS and SARS-CoV have also provided a high benefit for rapid designing of COVID-19 vaccine.

As per draft landscape of COVID -19 candidate vaccines published by the WHO on 22nd January 2021, 10 nonreplicating and 4 replicating (overall 14) viral vector–based vaccine candidates are under clinical evaluation. Nevertheless, a total of 39 are under preclinical trial, among which 20 are nonreplicating and 19 are replicating viral vectors. The vaccine development program for COVID-19 had used a wide spectrum of vectors including Ad, MVA, Sendai viruses, parainfluenza viruses, rabies viruses, influenza viruses, and Newcastle viruses. Intramuscular route is the preferable route of administration for most of the vaccine candidates. Four adenovirus–based vaccine candidates (ChAdOx1-S, adenovirus type 5 vector, adeno-based (rAd26 + rAd5-S), and Ad26CoVS_1_) have reached phase III of clinical trial (WHO, 2021). A complete list of viral vector–based COVID-19 vaccines under clinical trial is given in Table 1.

**TABLE 1:** List of viral vector–based COVID-19 vaccines under clinical trial (WHO, 2021).

Vaccine	Route	Phase	Sponsor(s)	Clinical trial ID
			Nonreplicating viral vector–based vaccine
AZD1222 (ChAdOx1-S)	IM	III	University of Oxford	ISRCTN89951424
			AstraZeneca	NCT04516746
				NCT04540393
			Serum Institute of India Pvt. Ltd	CTRI/2020/08/027170
Adenovirus type 5 vector	IM	III	CanSino Biological Inc	NCT04526990
			NPO Petrovax	NCT04540419
Gam-COVID-vac	IM	III	Gamaleya Research Institute	NCT04530396
				NCT04564716
Ad26.COV-S (JNJ-78436735, Ad26COVS1)	IM	III	Janssen Vaccines and Prevention B.V.	NCT04505722
				NCT04614948
hAd5-S-Fusion + N-ETSD	SC	I	ImmunityBio, Inc	NCT04591717
GRAd-COV2	IM	I	ReiThera Srl	NCT04528641
Ad5-nCoV	IM	I	Institute of Biotechnology, Academy of Military Medical Sciences, PLA China	NCT04552366
VXA-CoV2-1	Oral	I	Vaxart	NCT04563702
MVA-SARS-2-S	IM	I	University Medical Center Hamburg-Eppendorf	NCT04569383
AdCOVID	IN		Altimmune	NCT04679909
Replicating viral vector–based vaccine				
DelNS1-2019-nCoV-RBD-OPT1)	IN	II	Beijing Wantai Biological	ChiCTR2000039715
			Pharmacy and Xiamen University	
IIBR-100	IM	II	Israel Institute for Biological Research	NCT04608305
V590	IM	I	Merck Sharp and Dohme	NCT04569786
COVID-19–101	IM	I	Institute Pasteur	NCT04497298

^a^IM: intramuscular, IN: intranasal, and SC: subcutaneous.

### Vaccines Under Phase III Clinical Development

#### ChAdOx1-S

The ChAdOx1-S or ChAdOx1 nCoV-19 is a nonreplicating viral vector–based vaccine candidate for COVID-19 designed by the University of Oxford in collaboration with AstraZeneca (Ledford, 2020). It is presently known as AZD1222 and is presently undergoing phase III human trial. Upon the emergence of SARS-CoV-2, one of the promising vaccine candidates for MERS-CoV, ChAdOx1 MERS has been repurposed, and AZD1222 encoding a full-length codon–optimized S protein of SARS-CoV-2 has been designed. ChAdOx1 is an isolate Y25-derived replication-deficient simian adenoviral vector (Precision vaccination, 2020c). As reported by Dicks et al., in the human population the seroprevalence of antibodies to Y25 is 0% in the United Kingdom and 9% in Gambia (Dicks et al., 2012). Preclinical study in mice and rhesus macaques demonstrated that AZD1222 can elicit a strong cell-mediated and humoral immune response. As per the report, AZD1222 can induce a stable Th1/Th2 humoral and cellular response, and a significant reduction of viral load in the lower respiratory tract tissue and broncho alveolar lavage fluid as compared to control. Notably, no evidence of pneumonia and immune-enhanced diseases were detected in immunized animals (van Doremalen et al., 2020). A preliminary phase I/II study report of AZD1222 published on July 20, 2020, showed an acceptable safety profile, high immunogenicity and tolerability of the vaccine candidate. However, the reduction in reactogenicity was observed with paracetamol and following second dose. The study also observed a four-time increase in antibodies to S protein of SARS-CoV-2 in 95% of subjects, after one month of AZD1222 single dose injection (Folegatti et al., 2020). Clinical development of AZD1222 has progressed worldwide with late-stage phase II/III clinical trials in various countries including the United Kingdom (ISRCTN89951424), the United states (ClinicaltrialsNCT04516746, 2020e), Russia (ClinicaltrialsNCT04540393, 2020i), and India (ClinicaltrialsCTRI/2020/08/027170, 2020). In India, AZD122 is dubbed as Covishield (Times Now Digital, 2020). As of September 12, 2020, as part of the clinical trial, 18,000 individuals have received AZD1222 worldwide. As per AstraZeneca, following safety and efficacy assessment, 300 million doses of AZD1222 will be available by July 2021 (CEPI, 2020). A study report published on January 9th, 2021, confirmed the safety profile of AZD1222 and proved to be efficacious against symptomatic treatment of SARS-CoV-2 infection (Voysey et al., 2021).

#### Adenovirus Type 5 Vector Vaccine

CanSino Biological Inc. and Beijing Institute of Biotechnology are developing a adenovirus type 5 vector vaccine, which is a replication-defective adenovirus type-5 (Ad5) vector expressing the S glycoprotein of SARS-CoV-2 strain (Precision vaccination, 2020a; Zhu et al., 2020b) A non-randomized, single-centered, dose-escalation, open-label, phase I trial for adenovirus type 5 vector vaccine was performed on healthy adults (18–60 years) in Wuhan, China. The study results found adenovirus type 5 vector vaccine to be immunogenic and tolerable in healthy adults and can produce both humoral (at day 28 postvaccination (Precision vaccination, 2020b) and cellular (from day 14 after single dose) immunity against SARS-CoV-2. Among the three doses group, high dose of the vaccine shows more immunogenicity compared with the middle and low dose. However, the high dose was associated with higher reactivity, and some adverse effects such as severe fever, dyspnea, fatigue, joint pain, and muscle pain were also reported (Zhu et al., 2020b). Following this, a placebo-controlled, double-blind, randomized, phase II study was performed, which extended the knowledge of the immunogenicity and safety of the adenovirus type 5 vector vaccine. Older population showed higher tolerability but lower immune response than younger population and thus the study assumed that a supplementary dose might be required for older population. Except mild, transient vaccination–related adverse effects no serious adverse effects were detected. In majority of the recipients, immunization with single dose of the vaccine induced rapid onset of immunity within 14 days and significant humoral and cellular immunity within 28 days. For further evaluation of efficacy, this phase II trial support experimenting adenovirus type 5 vector vaccine at 5×10^10^ viral particles in a phase III effectiveness trial in healthy adults (Zhu et al., 2020a). Till date, two phase III clinical trial has been registered in *ClinicalTrials.gov*. A double-blind, placebo-controlled phase III study sponsored by CanSino Biologics Inc. has started on 15 September 2020 with the aim to evaluate immunogenicity, safety, and efficacy of adenovirus type 5 vector vaccine in healthy volunteers (18 years or above) (ClinicaltrialsNCT04526990, 2020f). Another randomized, double-blind phase III trial started on 11 September 2020 was sponsored by NPO petrovax, that will assess the efficacy, safety, and reactogenicity of the vaccine compared with placebo in total of 500 healthy subjects (age from 18 to 85 years) (ClinicaltrialsNCT04540419, 2020j).

#### Gam-COVID-VacLyo

The vaccine candidate Gam-COVID-VacLyo is being developed by Gamaleya Research Institute which consists of two recombinant adenovirus vector components, type 26 (rAD26) and type 5 (rAd5), and both encoding with S glycoprotein of SARS-CoV-2 virus (rAD26-S and rAD5-S). Safety and immunogenicity assessment of lyophilized and frozen formulation of this vaccine was examined in two phase I/II clinical trials on healthy adults (18–60 years aged) at two Russian hospitals. Safety of the two individual components of the vaccine candidate Gam-COVID-VacLyo was established in phase I trial. Then, in phase II, as a prime-boost vaccination, both components were injected intramuscularly and it showed that the vaccine had a good tolerability and generate strong cellular and humoral immune responses in the subjects. Moreover, antibody titers were higher in vaccinated subjects than those in convalescent plasma. Postvaccination antibodies were increased significantly from day 14 and cellular immunity was peaked at day 28. Additionally, the vaccine did not cause any serious adverse events in healthy adult participants (Logunov et al., 2020). Recently, an open-ended prospective non-randomized phase II study has started with 110 volunteers over the age of 60, with the goal to assess tolerability, safety, and immunogenicity of Gam-COVID-VacLyo (ClinicaltrialsNCT04587219, 2020q). On September 7, 2020, a phase III clinical trial has been started with the official title “Randomized Double-Blind Placebo-Controlled Multi-Center Clinical Trial in Parallel Assignment of Efficacy, Safety, and Immunogenecity of Gam-COVID-Vac Combined Vector Vaccine in SARS-CoV-2 Infection Prophylactic Treatment.” This trial will involve a total of 40,000 participants over the age of 18 years, and they will be randomized (3:1) into placebo receiving reference group (10,000 subjects) and test group (30,000 subjects). This study has been sponsored by Gamaleya Research Institute in collaboration with Government of the City of Moscow and CRO: Crocus Medical BV (ClinicaltrialsNCT04530396, 2020h). Another phase III trial has started on September 28, 2020, in Belarus, which has also been sponsored by Gamaleya Research Institute in collaboration with Russian Direct Investment Fund and CRO: iPharma; it is a double-blind, multicenter, placebo controlled randomized, phase III trial for assessment of efficacy, immunogenicity, and safety of the vaccine candidate Gam-COVID-VacLyo against COVID-19 infection. The study will include a total of 110 volunteers between the age of 18–60 (ClinicaltrialsNCT04564716, 2020n). Furthermore, the UAE and Venezuela had started phase III trial to determine the safety, immunogenicity, and efficacy of this vaccine candidate (ClinicaltrialsNCT04642339, 2020u; ClinicaltrialsNCT04656613, 2020v).

#### Ad26.COV2-S

Janssen Pharmaceutical Companies has nominated their main vaccine candidate recombinant Ad26.COV2-S (Ad26COVS1/JNJ-78436,735) for prophylaxis of COVID-19 infection. A phase I/IIa, placebo-controlled, randomized, double-blind trial is in progress since July 15, 2020, with the aim of assessing safety, immunogenicity, and reactogenicity of Ad26.COV2-S against SARS-CoV-2 in healthy adults (18–55 years old) (ClinicaltrialsNCT04436276, 2020a). On July 30, 2020, a study report published in *Nature* confirmed that a single dose of Ad26.COV2-S provoked robust neutralizing antibody titers, fruitfully averting subsequent infections and provided wide-range protection against SARS-CoV-2 in both the upper and lower respiratory tract of rhesus macaques (Mercado et al., 2020). Another study published on September 3, 2020, established that a single dose of Ad26.COV2-S can protect hamsters against the clinical conditions resulting after a high-dose intranasal challenge of SARS-CoV-2; it also elicited binding and neutralizing antibody response (Tostanoski et al., 2020). To evaluate the safety and efficacy of Ad26.COV2-S, a large-scale, randomized, pivotal, double-blind, multicentric, placebo-controlled phase III trial (ENSEMBLE) has been launched by the developer companies, which will enroll up to 60,000 volunteers across three continents (ClinicaltrialsNCT04505722, 2020d). A non–peer-reviewed research report established that a single immunization of Ad26.COV2-S is safe and immunogenic against SARS-CoV-2 (Sadoff et al., 2020). Nevertheless, recently ENSEMBLE-2, another phase III trial has been started for the efficacy and safety assessment of the vaccine (ClinicaltrialsNCT04614948, 2020t).

Phase I clinical trials are in progress for five more nonreplicating viral vector–based vaccine candidates.

The Ad5-S-Fusion + N-ETSD is a vaccine candidate sponsored by ImmunityBio, Inc., which is a bivalent human adenovirus serotype 5 (hAd5) vector with E1/E2b/E3 omissions and expressing spike glycoprotein of SARS-CoV-2 and a conserved nucleocapsid (N) with an improved T-cell stimulation domain. This vaccine candidate was suggested to be optimized for immunogenicity, as per a preclinical study report, because the S-fusion shows enhanced S receptor–binding domain (RBD) cell surface expression, which reserved conformational integrity and identification by ACE2-Fc. The N-ETSD protein is restricted to lysosomal/endosomal subcellular compartments for MHC I/II presentation. Refinements on S-Fusion and N-ETSD had enhanced *de novo* humoral and cellular immune response in antigen-naive preclinical models (Rice et al., 2020). Based on preclinical indications, ImmunityBio, Inc. has started a phase Ib, open-label study in healthy adults, where safety, immunogenicity, and reactogenicity of the vaccine candidate will the assessed (ClinicaltrialsNCT04591717, 2020r). Moreover, an open-label, phase Ib trial has been designed in South Africa to confirm the safety profile and to determine a proper dose of the vaccine for further studies (ClinicaltrialsNCT04710303).

The GRAd-COV2 is a vaccine candidate against COVID-19 pandemic based on a novel replication-incompetent simian adenovirus strain expressing the full-length S glycoprotein of SARA-CoV-2, which is being developed by ReiThera, an Italian–based biotech company. Simian adenoviral vectors represent an extensively used vaccine against many emerging infectious diseases because of their excellent safety profile, advanced manufacturing methods, and rapid onset of cellular and humoral immune responses (covidX, 2020). A phase I, dose escalation open-label, multicenter clinical trial (RT-CoV-2) is ongoing to determine the safety and immunogenicity of GRAd-COV2 against SARS-CoV-2 (ClinicaltrialsNCT04528641, 2020g).

One more recombinant adenovirus type 5 vector vaccine, namely, Ad5-nCoV is in phase I clinical trial sponsored by the Institute of Biotechnology, Academy of Military Medical Sciences, PLA of China. The study will perform safety and immunogenicity assessment of two doses of Ad5-nCoV followed by mucosal and intramuscular vaccination in different administration schedules (ClinicaltrialsNCT04552366, 2020k). The VXA-CoV2-1 is an oral recombinant adenoviral-vector based vaccine expressing an antigen of SARS-CoV-2 and dsRNA adjuvant. VXA-CoV2-1 is formulated by Vaxart (an American biotech. Company), as an enterically coated tablet. The vaccines target the small bowel, thus engaging the finely tuned immune system of the gut to produce broad, persistent systemic and mucosal immune response (NCT04563702; VAXART, 2020). Vaxart has designed an open-label, dose-ranging phase I clinical study for safety and immunogenicity assessment of two doses of VXA-CoV-1 against SARS-CoV-2 in healthy adult subjects (aged 18–54 years) (ClinicaltrialsNCT04563702, 2020m). MVA-SARS-2-S is another promising COVID-19 vaccine candidate, which comprises a modified vaccinia virus Ankara (MVA) vector-expressing S protein of SARS-CoV-2. Recombinant MVA vector has revealed promising results in MERS-CoV vaccine development (Koch et al., 2020). Depending on this, two MVA-based vaccines MVA/S (express a prefusion state stabilized, membrane–anchored full-length S protein) and MVA/S1 (express the S1 region of the spike which forms trimers) had developed during COVID-19 pandemic, and a preclinical study on the mouse model had demonstrated MVA/S as a promising vaccine candidate against SARS-CoV-2 (Routhu et al., 2020). To determine the safety, tolerability, and immunogenicity of MVA-SARS-2-S, an open, single-center phase I trial has been developed by University Medical Center Hamburg-Eppendorf. The study includes healthy subjects in two different dose cohorts and vaccinated twice with the proposed vaccine candidate (ClinicaltrialsNCT04569383, 2020o).

Alongside nonreplicating viral vector–based vaccine candidates, replicating viral vector–based vaccine candidates are also in headway.

The vaccine candidate DELNS1-2019-nCoV-RBD-OPT1 is established on flu-based DelNS1 live-attenuated influenza virus (LAIV) platform from which immune antagonist (NS1) and the key virulent element has been deleted and encoded with SARS-CoV-2’s S protein. DELNS1-2019-nCoV-RBD-OPT1 has been developed as an intranasal spray and is being evaluated in China for its safety and immunogenicity (ChiCTR2000037782, 2020). Recently, a phase II clinical trial has been started for this vaccine candidate (ChiCTR2000039715, 2020).

Israel Institute of Biological Research (IIBR) has developed a replication-competent vesicular stomatitis virus (VSV) vaccine candidate, encoding S protein of SARS-CoV-2 (rVSV-SARS-CoV-2-S). Preclinical investigation revealed that, rVSV-SARS-CoV-2-S resembles the SARS-CoV-2, in antigenicity, spike expression properties, and neutralizing antibody production ability. Furthermore, single-dose immunization with the vaccine candidate, proved to be safe, effective, and elicited sufficient neutralizing antibody against SARS-CoV-2 infection (Yahalom-Ronen et al., 2020). Another study by Li et al., 2020b concluded that rVSVs holds excellent potential for studying SASR-CoV-2 host interactions, immune response characterization, and neutralizing antibodies detection and can be a promising vaccine candidate for prophylaxis of COVID-19 (H. Li et al., 2020). Additionally, Dieterle et al., 2020 reported that, in high-throughput fluorescent reporter assay, the neutralization properties of a huge panel convalescent sera of COVID-19 can be evaluated with rVSV-SARS-CoV-2S and the antisera neutralization of the rVSV and SARS-CoV-2 is vastly correlated (Dieterle et al., 2020). Considering these results, a phase I clinical trial has been started to evaluate safety, efficacy, and immunogenicity of rVSV-SARS-CoV-2S in healthy adults, and the study has been promoted to phase II on 28th October 2020 (ClinicaltrialsNCT04608305, 2020s).

Another vaccine candidate, V590 is a recombinant vesicular stomatitis virus (rVSV) developed by Merck and Co., which is repurposed from Merck’s Ebola Zaire virus vaccine (GEN, 2020). A phase I study has begun with the hypothesis that as determined by plaque reduction neutralization test, so far, a possible one well-tolerated dose of V590 raises the geometric mean titers of COVID-19 anti-serum neutralizing antibody when compared to placebo (ClinicaltrialsNCT04569786, 2020p). Furthermore, a phase I randomized, placebo-controlled, two center trial on a novel measles vector-based vaccine candidate, developed by Institut Pasteur, is in progress to determine its safety, tolerability, and immunogenicity against SARS-CoV-2 (ClinicaltrialsNCT04497298, 2020b). Furthermore, a phase I/phase II trial has been started with the objective to identify the dose range in order to reach targeted immunogenicity (ClinicaltrialsNCT04498247, 2020c). An intranasal adenovirus type 5 (Ad5) vector vaccine candidate (AdCOVID) expressing the RBD of the S protein of SARS-CoV-2, had shown a potent immune response against RBD *via* the generation of serum neutralizing antibodies, mucosal IgA induction, and expression of CD4^+^ and CD8^+^ T-cells with a Th-1–like cytokine (King et al., 2020). A phase I trial has been designed by Altimmune to assess the safety and immunogenicity of AdCOVID (ClinicaltrialsNCT04679909, 2020w).

Accompanying the clinical trials, 39 preclinical studies on viral vector–based COVID-19 vaccine are in progress and are showing promising results. Table 2 summarizes the list of viral vector–based COVID-19 vaccine candidates undergoing preclinical evaluation (WHO, 2021).

**TABLE 2:** List of viral vector–based vaccine candidates under preclinical evaluation (WHO, 2021).

Developer/manufacturer	Type of vaccine candidate
Nonreplicating viral vector–based vaccine candidates	
University of Helsinki/University of Eastern Finland	Ad 5 vector
Theravectys-Institut Pasteur	Lentiviral vector
ID Pharma	Sendai virus vector
Ankara University	Adenovirus-based
Massachusetts General Hospital/AveXis	AAVCOVID
GeoVax/BravoVax	MVA-encoded VLP
DZIF- German Center for Infection Research/DT Biologika GmbH	MVA-S encoded
IDIBAPS-Hospital Clinic (Spain)	MVA-S
AIOVA	Lentiviral vector
Erciyes University	Adeno5-based
Greffex	Ad5 S
Stabilitech Biopharma Ltd	Oral Ad5 S
Valo Therapeutics Ltd	Adenovirus-based + HLA-matched peptides
Centro Nacional Biotecnologia (CNB-CSIC) (Spain)	MVA expressing structural proteins
University of Georgia/University of Lowa	PIV5
Bharat Biotech/Thomas Jefferson University	Recombinant deactivated rabies virus containing S1
National Research Center (Egypt)	Influenza a H1N1 vector
Icahn School of Medicine at Mount Sinai	Newcastle disease virus expressing S
Vaxart	Oral vaccine platform
Sorbonne University	Lentiviral vector Retro-VLP particles
Replicating viral vector–based vaccine candidates	
Farmacologicos Veterinarios SAC (FARVET SAC)/Universidad Peruana Cayetano Heredia (UPCH)	rNDV-FARVET expressing RBD
KU Leuven	YF17D vector
Cadila Healthcare Limited	Measles vector
FBRI SRS VB VECTOR, Rospotrebnadzor, Koltsovo	Measles vector
CanVirex AG/DZIF- German Center for Infection Research	Measles virus (S, N targets)
Tonix Pharma/Southern Research	Horsepox vector expressing S protein
BiOCAD/IEM	Attenuated influenza virus based live viral vectored vaccine
FBRI SRC VB VECTOR, rospotrebnadzor, Koltsovo	Influenza–based recombinant vaccine
Instituto Buntantan/Fundacao Oswaldo Cruz	Attenuated influenza expressing an antigenic portion of the spike protein
University of Hong Kong	Influenza vector expressing RBD
University of Manitoba	Replicating VSV vector–based DC-targeting
University of Western Ontario	VSV-S
Aurobindo	VSV-S
FBRI SRC VB VECTOR, Rospotrebnadzor, Koltsovo	VSV vector
UW-Madison, Bharat Biotech and FluGen	M2SR influenza vector
Intravacc, Utrecht University, Wageningen Bioveterinary Research	NDV-SARS-CoV-2/Spike
The Lancaster University (United Kingdom)	APMV
Farvet SAC	rNDV-LS1-HN-RBD/SARS-CoV-2
Farvet SAC	rNDV-LS1-S1-F/SARS-CoV-2

## Conclusion and Future Prospective

The outbreaks of SARS in 2002, MERS in 2012, and COVID-19 in 2019 show that there has been a new major CoV outbreak in every decade of the 21st century so far. Thus, it can be expected that such epidemics can emerge in the future. However, in order to contain the prevailing scenario of COVID-19, rapid development of a safe, reliable, and potent vaccine is urgently needed. An ideal vaccine should have the ability to generate excellent immunogenic response (irrespective of age) with low antigen dosage against different viral strains of the same pathogen with no adverse effects. Although several attempts have been made in order to generate a successful vaccine candidate for the prophylaxis of SARS-CoV-2, till date, no approved vaccine is available. The foremost challenge while developing a vaccine against newly emerging virus is to find out various properties of the virus such as mode of entry, target organ, genomic sequence, mechanism of action, mutation, development of immunity, symptomatic and asymptomatic nature, and relapse of the infection. Similarly, while using viral vectors as platform for vaccine development, it is necessary to identify the genotoxicity, epidemiology, and virology of pathogenic as well as viral vector viruses. Thus, for the newly emergent viruses like SARS-CoV-2, rapid production of a viral vector–based vaccine is difficult. Moreover, delay of the real anticipated immune response to the pathogenic virus is also a significant restriction in case of viral vector–based vaccine candidates, because preexisting immune response is primarily acquired due to the vector virus.

Importantly, common challenges with all COVID-19 vaccine platforms include unavailability of the adequate preclinical model and establishment of a proper administration route. Selection of the animal model for preclinical testing of vaccine itself is associated with several challenges such as presence of the natural immunity against SARS-CoV-2, absence of ACE2 receptor, and unanticipated pathogenicity or immunogenicity against the testing virus.

Nevertheless, another major issue, particularly, in manufacturing of vaccine candidate is the bioprocessing scale-up of the vaccine with the highly pure antigen. Currently all the experiments on vaccine candidates are achieved by small-scale production of antigen in the laboratory; however, in the race for rapid production of vaccine, large-scale production might be hampered by the purity of the antigen product. Thus, safety and immunogenicity of the vaccine candidate may be altered and may lead to some untoward adverse effects.

Though there are many challenges associated with the production of vaccine against COVID-19 infection, with a growing number of research studies progressing toward the later stage of clinical trials, there is tremendous enthusiasm and optimism in the area of viral vector–based vaccine development against the pandemic. For instance, ChAdOx-S is showing an excellent potential as a promising COVID-19 vaccine candidate, 2020. On September 6, 2020, AstraZeneca, the ChAdOx-S developing company, had paused the trials after a woman participant showed some neurological symptoms related to transverse myelitis. However, the trial resumed in 2020, after confirming the safety of the vaccine candidate by the UK’s Medicines Health Regulatory Authority (Live Science, 2020; STAT, 2020). The U.S. Department of Health and Human Services (HHS) had announced up to $1.2 billion to AstraZeneca for rapid development of the vaccine and to manufacture at least 300 million doses, after assessment of safety and efficacy of the vaccine candidate (Division, 2020). Furthermore, Johnson & Johnson had an agreement of $1 billion, with the U.S. government to deliver 100 million doses of the vaccine Ad26.COV2.S in the U.S., following its approval or authorization for emergency use from the U.S. FDA (Johnson and Johnson, 2020). Therefore, considering the preclinical and clinical progress of viral vector–based vaccine candidates in the race of rapid vaccine development, it can be assumed that till the end of this year, a safe and effective viral vector–based vaccine will be available to curtail the COVID-19 pandemic. However, various international funding agencies should mobilize and come forward to promote the vaccine development program by overcoming various challenges and for stockpiling of COVID-19 vaccine.

## Abbreviations

AAV, Adeno-associated virus; ACE2, Angiotensin converting enzyme receptor 2; Ad, Adenovirus; AIDS, Acquired immunodeficiency syndrome; COVID-19, Coronavirus disease 2019; EMA, European Medical Agency; FDA, Food and Drug Administration; HA, Hemagglutinin; HIV, Human immunodeficiency virus; HN, Hemagglutinin-neuraminidase; hPIV-1, Human parainfluenza type-1 virus; IFN, Interferon; ITR, Inverted terminal repeats; kb, kilobase; LAIV, Live-attenuated influenza virus; MERS-CoV, Middle East respiratory syndrome coronavirus; MV, Measles virus; MVA, Modified vaccinia virus Ankara; NP, Nucleoprotein; YVAC, New York–attenuated vaccinia virus; PbCS, Circumsporozoite antigen of; *Plasmodium berghei*; PfCS, Circumsporozoite antigen of *Plasmodium falciparum*; PROSTVAC, Poxviral prostate–specific Ag-targeting vaccine; R&D, Research and Development; rMV, Recombinant measles viruses; S proteins, Spike proteins; SARS-CoV, Severe acute respiratory syndrome coronavirus; SARS-CoV-2, Severe acute respiratory syndrome coronavirus 2; SeV, Sendai virus; TAAs, Tumor-associated antigens; TLR, Toll-like receptors; TSAs, Tumor-specific antigens; VACV, Vaccinia virus; VSV, Vesicular stomatitis virus; WHO, World Health Organization.
